# Assessing the Impact of On-Farm Biosecurity Coaching on Farmer Perception and Farm Biosecurity Status in Belgian Poultry Production

**DOI:** 10.3390/ani14172498

**Published:** 2024-08-28

**Authors:** Arthi Amalraj, Hilde Van Meirhaeghe, Ilias Chantziaras, Jeroen Dewulf

**Affiliations:** 1 Unit of Veterinary Epidemiology, Faculty of Veterinary Medicine, Ghent University, Salisburylaan 133, 9820 Merelbeke, Belgium; ilias.chantziaras@ugent.be (I.C.); jeroen.dewulf@ugent.be (J.D.); 2 Vetworks BV, Knokstraat 36, 9880 Aalter, Belgium; hilde.vanmeirhaeghe@vetworks.eu

**Keywords:** ADKAR^®^ model, Belgium, biosecurity, coaching, poultry

## Abstract

**Simple Summary:**

Effective communication between farmers and veterinarians enables the careful assessment of biosecurity aspects, often leading to mutual agreement over on-farm changes. Coaching is a learner-driven process in which the coach is not simply providing advice or direction but rather enabling the learner to identify a goal and find solutions. Farmers’ readiness to accept changes is pivotal to biosecurity applications, and the recognition of the barriers to change is highlighted in the study. Numerous farmers in this study have embraced the action plan, indicating that offering tailored guidance through direct face-to-face coaching in a few specific areas has the potential to be an effective approach for disseminating biosecurity information. The farmers showed a positive response to the individualized coaching, particularly considering that it came from individuals who were not their usual source of biosecurity information. The authors have found success in enhancing farm biosecurity procedures with coaching using the two complementary scoring tools ADKAR^®^ (Mont-Saint-Guibert, Belgium) and Biocheck.UGent^TM^ (Dentergem, Belgium).

**Abstract:**

Veterinary coaching was tested to assess its efficacy in promoting adherence to biosecurity procedures. Poultry farmers (*n* = 13) in Belgium were profiled using ADKAR^®^, coached and audited prior to and 6 months after coaching. The ADKAR^®^ (Awareness, Desire, Knowledge, Ability, and Reinforcement) profiling technique identified 5/13 participating farmers with relatively low scores (≤3) for one or more elements that block change (biosecurity compliance in this case). Education was the only demographic variable that influenced knowledge scores. Through the Biocheck.Ugent^TM^ methodology, farm biosecurity was assessed and benchmarked to allow for tailored guidance. The farmer, farm veterinarian, and coach defined a farm-specific action plan that covered infrastructure, site access, staff/visitors, purchase policies, transport and depopulation, feed and water supplies, flock management, cleaning and disinfection between flocks, and measures between houses. From a total of 49 proposed actions, 36 were adopted. Purchasing policy had the highest (100%) and cleaning and disinfection had the lowest compliance (38%). Time, cost, and feasibility (e.g., inadequate farm layout) were the main reasons cited for not implementing action points. Overall, biosecurity improved significantly (*p* = 0.002) from 67.1 ± 5.7% to 70.3 ± 5.7% (mean ± Std. dev). The study, hence, presents convincing proof of how coaching can lead to new solutions not previously considered.

## 1. Introduction

Farm biosecurity is a combination of all measures applied to prevent the introduction and spread of infections on a farm [1] and is a very important component in the protection against infectious diseases, especially for livestock farmed under conventional modern production systems. It has been shown that training farm staff on biosecurity practices contributes to improving biosecurity [2,3], particularly by taking into account their perspectives and current practices, as well as other issues such as language barriers, educational gaps, and prior job experience [4]. This illustrates that a practical and people-centered approach is needed to help undertake biosecurity measures [5]. In human medicine [6,7,8] and veterinary practice [9,10,11], compliance strategies like verbal and written warnings, training programs, audits, and even surveillance methods such as cameras have been shown to be effective in improving adherence to biosecurity protocols. 

Farmers’ readiness to adopt new practices is vital for enhancing biosecurity, since such measures involve daily routine changes, the results of which are not always immediately apparent [12]. As a result, enhancing biosecurity necessitates changes in farmers’ attitudes and actions. Coaching may be beneficial in achieving long-term behavioral changes [13], where a nondirective questioning strategy helps the learner identify a goal and find solutions [14]. Because farmers are more likely to take action based on information that is customized to their specific circumstances [15], one-on-one communication (e.g., coaching) allows for tailored guidance to align with the farmers’ needs [16]. In fact, personalized coaching has been shown to be effective in poultry production [10,13]. However, to guarantee a more successful biosecurity commitment, the coach’s expertise, the amount of time allocated, and an understanding of farm staff attitudes and compliance are essential for such unstructured trainings [17]. Moreover, not many studies assessed adherence to biosecurity practices and lasting behavior changes [18,19]. Therefore, the change management model ADKAR^®^, an acronym for the five elements necessary for successful change—Awareness, Desire, Knowledge, Ability, and Reinforcement—was adapted and used to understand the attitudes and behaviors of different kinds of poultry producers [20,21]. On-farm risks were evaluated using the Biocheck.UGent^TM^ scoring system (https://biocheckgent.com/en, accessed on 27 July 2022), which has the benefit of offering an objective and comprehensive assessment of biosecurity at the farm level.

The purpose of this study was to determine how much of an improvement in farm biosecurity and attitudes toward biosecurity implementation could be achieved through on-farm coaching. The second objective was to find out how these improvements could then be linked to the farmers’ ADKAR^®^ profiles. This study is centered on change management under the assumption that the successful management of poultry farm operations depends on timely decision making by farmers.

## 2. Materials and Methods

### 2.1. Study Design

**Figure 1:**
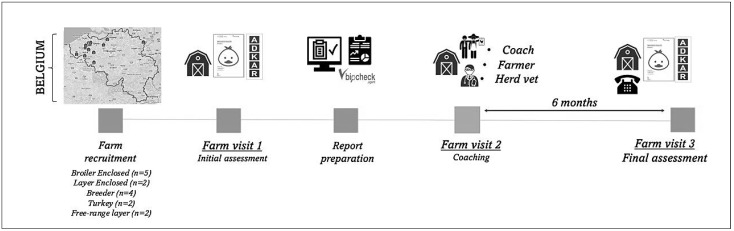
On-farm biosecurity coaching in Belgian poultry farms: a longitudinal study.

A longitudinal study was performed on 15 poultry farms (broiler, *n* = 5; layer, *n* = 2; breeders, *n* = 4; turkey, *n* = 2; and free-range layer, *n* = 2) in Belgium between June 2022 and June 2023. The participating farms were visited on three occasions during the 12-month period, and data were collected during these farm visits (Figure 1). During the first farm visit, data on biosecurity levels and farmers’ attitudes towards biosecurity were assessed by the coach. The project’s assigned “facilitator” handled the planning remotely, while the assigned “coach”, in consultation with the herd veterinarian, organized and carried out the second farm visit. The visit included a face-to-face interaction with the farmer, and together with the herd veterinarian, the positive aspects of the farm and areas for improvement were reviewed.

Based on the Biocheck.UGent^TM^ reports (see Section 2.4 coaching methodology), a farm-specific action plan was developed together with the farmer. An action plan, drawn in agreement with the farmers, documented every area where the farmer could and would improve over the next six months. The coach made follow-up phone calls with the veterinarian and farmer during the 6-month period (between visits 2 and 3) to monitor the progress of the implemented procedures, and any issues concerning the newly implemented measures were discussed. During the third visit, after a period of 6 months, data were again collected on the biosecurity levels and farm characteristics, and once again, the farmers’ attitudes towards biosecurity were profiled using the ADKAR^®^ model [21]. The designated “coach” conducting each farm visit was trained by the Netpoulsafe consortium in coaching techniques and how to perform the Biocheck.UGent^TM^ audit and interpret the biosecurity questionnaire. Improvements in biosecurity were recorded, and the facilitator and coach were contacted frequently to discuss the advancements and end results. 

A research team designed and facilitated the coaching process. This included a person trained in coaching who conducted the Biocheck.Ugent^TM^ audit and analyzed the sociological perspectives (H.V.M.), specialists in biosecurity issues in livestock farming (J.D. and I.C), and a third researcher who was part of the auditing team and was responsible for ensuring the proper implementation of this procedure (A.A.).

### 2.2. Farm Selection and Recruitment

The inclusion of farms was based on the following criteria: (1) for the farm type, poultry farms needed to be conventional enclosed broiler, enclosed layer, parent breeding, turkey, or free-range layer companies; (2) farmers’ readiness to take part throughout the study period, which included farmer profiling, farm biosecurity evaluations, coaching, and biosecurity application. Because of the nature of the study and the limited number of farms that needed to be recruited, the professional connections of the authors facilitated the recruiting process. All participating farmers were informed of the study’s purpose prior to enrollment. In order to promote long-term commitment and improve compliance with the action plan, herd veterinarians were involved as much as possible.

### 2.3. Farmer Data Collection

**Figure 2:**
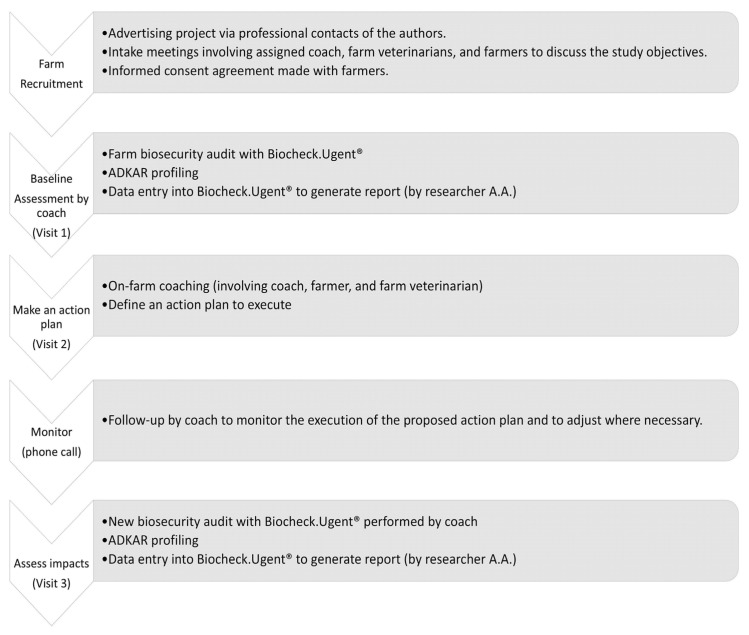
Overview of steps in the validation of the selected supporting measure “on-farm coaching”.

Questions relating to participating farmers’ genders, ages, educational qualifications, years of work experience in poultry farming, job satisfaction, and number of employees were asked. The farmers were assured that any information that may be used to identify them would be anonymized. Every farmer gave signed consent to data collection, data management, storage, participation in coaching and follow-up, and results dissemination. Figure 2 outlines the various steps adopted.

#### 2.3.1. Quantification of Farm Biosecurity

The biosecurity status was quantified using the Biocheck.UGent™ questionnaires (Supplementary File), a risk-based scoring system [22] used in numerous studies related to broiler and layer production [3,22,23,24,25,26,27,28,29]. The poultry broiler and layer tools remain limited to the specific production contexts in which they were developed. To measure biosecurity in a standardized manner in breeder, turkey, and free-range layer production, the authors, in conjunction with poultry experts across Europe, previously developed species-specific systems for poultry [30] that are comparable to the broiler and layer surveys. This allowed for a better assessment of the on-farm risks and provided a standard benchmark for helping the producers achieve and maintain a good biosecurity status. Prior to completing the online Biocheck.UGent™ questionnaire “https://biocheckgent.com/en (accessed on 27 July 2022)”, the farms were visited, and visual observations were made (such as observing the entrance design, locations of nearby water bodies, biosecurity protocols in place, and so on) to avoid answers that the farmer wished to report but did not hold true. The auditing team included two authors with experience in poultry farm biosecurity. 

#### 2.3.2. ADKAR^®^ Profiling of Farmers

To explore farmers’ attitudes towards recommended biosecurity practices and the factors that block compliance, we used a behavioral change model called ADKAR^®^, an acronym for the five elements necessary for successful change: Awareness, Desire, Knowledge, Ability, and Reinforcement. The authors adopted and applied the ADKAR^®^ change management methodology [31], a widely recognized and valuable method used in the fields of corporate business and human [32] and veterinary medicine [20,21,33]. By following the guidelines outlined by Amalraj et al. [21], the farmers were profiled, and scores were assigned to each element after asking the following questions:

For awareness: Are you familiar with the term biosecurity? Do you consider the current biosecurity measures to be adequate?

For desire: Are you satisfied with the measures or actions you have already implemented on your farm? Do you want to implement any new measures?

For knowledge: What are the different risks that threaten your farm? What are the weaknesses regarding poultry health?

For ability: What actions will you take to deal with the problem? What is stopping you from making a plan?

Each ADKAR element was given a score between 1 and 5, with 1 denoting the biggest obstacles to change [31]. Having a farmer-specific profile from visit 1 provided a foundation for the coaching sessions. The reinforcement (R) element was not assessed on visit 1, since no coaching or follow-up actions had been taken at that moment. On visit 3, a structured interview was conducted once again to profile the farmer’s views towards the adoption or non-adoption of the action plan.

### 2.4. Coaching Methodology

The goal of visit 2 was to co-create and co-own an action plan to increase the likelihood of implementing the proposed changes. To accomplish this, first, the designated coach presented each participant with benchmarked reports of the Biocheck.UGent^TM^ survey that was filled out during visit 1. Visit 2 entailed a face-to-face interview with both the farmer and veterinarian, and an unstructured facilitation technique was used to address the biosecurity strengths and weaknesses of the farm. The coach has already recognized the motivational barriers using the ADKAR profiles, which show that the acceptance of any change will be influenced by a score of 3 or less [21]. When farmers scored low (≤3) in awareness regarding biosecurity compliance, the focus was on addressing the “why” question in order to accomplish change. This was achieved by presenting information and examples on the dangers of poor biosecurity measures, as well as why change is necessary. For farmers who scored high on awareness from the start, the “why” question was only briefly addressed. Farmers with low “desire” scores (≤3) needed to be motivated to make changes by outlining and explaining the benefits associated with the proposed changes. Some farmers who desire to change may need help with the “how” aspect (low knowledge scores). This needed to be addressed by offering coaching tailored to their needs and circumstances. For the element “ability”, lower scores were dealt with by discussing topics such as making structural changes and investments towards better biosecurity. While the element reinforcement “R” was assessed once at six months of coaching, there was no opportunity to follow up on the positive improvements made due to the study’s completion. 

### 2.5. Data Analysis

Assumptions of normality for external, internal, and total biosecurity scores and attitude scores were tested through visual inspection of the Q–Q plot and the Shapiro–Wilk test. The different demographic variables (i.e., age, gender, and education) and the attitude scores for four elements (awareness, desire, knowledge, and ability) were not normally distributed. When the normality assumptions were met, an analysis of variance (ANOVA) test was used to analyze the impact of demographic variables on biosecurity and attitude scores. A parametric paired *t*-test was utilized to analyze potential differences in the biosecurity scores assessed with Biocheck.Ugent^TM^ before and after coaching. The non-parametric Kruskal–Wallis test and Mann–Whitney U test were performed when the variables did not seem normally distributed (based on a Shapiro–Wilk test). All statistical analyses were performed using SPSS^®^ Statistics Version 24 (IBM Corp., Armonk, NY, USA) and data management using Microsoft Excel. A *p*-value of <0.05 was considered significant. 

## 3. Results

### 3.1. Farm and Farmer Characteristics

**Figure 3:**
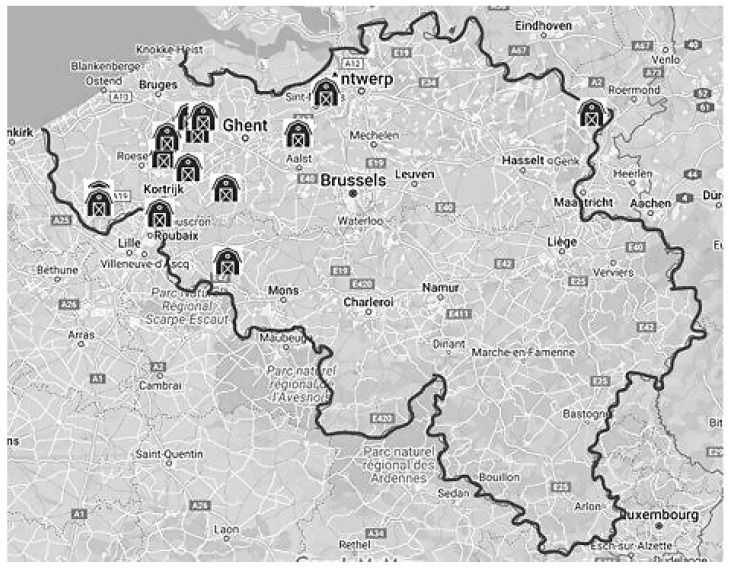
Map of Belgium with the geographical distribution of the study farms.

A total of 15 farms were enrolled in the study, but 2 (1 enclosed broiler and 1 free-range layer) were lost at follow-up. The reasons for discontinuing were (1) recurring flock health problems that impacted the motivation to continue and (2) no time. Those farms and farmers were not replaced, and the farms’ data are not presented. With the exclusion of two farmers, complete pre- and post-coaching biosecurity and attitude assessments collected from 13 farmers remained in the final analysis. The farmers of four enclosed broiler, two enclosed layer, four broiler breeder, two turkey, and one free-range layer farm took part in the study. Of the 13 enrolled farmers, 12 were from the Flemish-speaking region of Flanders, and 1 farmer was from the French-speaking region of Wallonia (Figure 3). The average (standard deviation) farm size was 57,295.8 (47,840.7) birds per farm (median = 39,750; range = 6500 to 150,000). Descriptive information regarding the demographical variables of education, age group, and gender associated with the participating farmers is presented in Table 1. Women represented 23.1% of the participating farmers (*n* = 3), and 76.9% (*n* = 10) were men. The majority of respondents were above 50 years of age (53.8%, *n* = 7/13), followed by middle-aged farmers between 35 and 50 years (23.1%, *n* = 3) and young farmers < 35 years of age (23.1%, *n* = 3). 

Most participating farmers attended up to secondary school (vocational) (84.6%, *n* = 10). They had on average (±Std. dev) 20.3 ± 13.4 years of experience in poultry production (median = 20; range = 2–44 years). Each farm employed on average (±Std. dev) 2.3 ± 1.2 staff (median = 2; range = 1–5). Among them were farmers owners, farm managers, family members, and other workers. Six farms (46.2%) had family members involved in the daily farm tasks. The majority of participating farmers were owners of their farm (92.3%). All 13 farmers reported good job satisfaction with their work.

### 3.2. Biosecurity

The external biosecurity status at the initial farm visit was, on average, 65.9% (range 56–81), and internal biosecurity was higher, with a score of 70.5% (range 53–87). The internal biosecurity scores differed significantly (*p* = 0.018) across production types, with the lowest scores obtained by the enclosed broiler (61.8%) and enclosed layer (61.0%) and the highest score obtained by the free-range layer (87.0%) farm (Table 1). No significant association was found between biosecurity and any of the observed demographic variables (*p* > 0.05). 

### 3.3. ADKAR^®^ Profiles 

**Figure 4:**
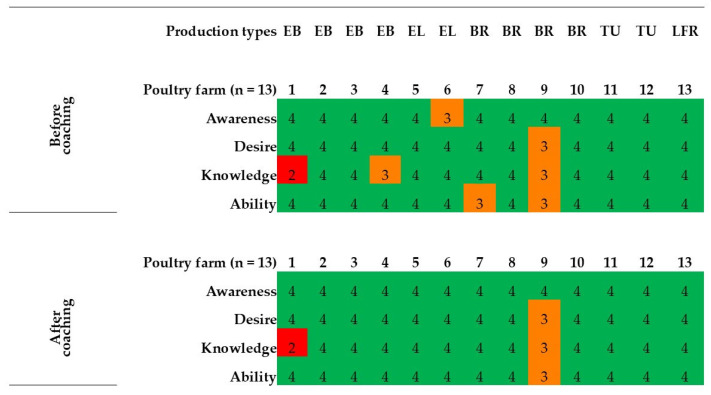
Individual ADKAR^®^ profiles of poultry growers (*n* = 13) for the elements Awareness, Desire, Knowledge, and Ability. A score of 1 denoted the lowest possible score, while a score of 5 denoted the greatest possible score. According to [31], if an element received a score of 1, 2, or 3, this element is likely to block the change or farmer’s intention towards biosecurity compliance. Poultry production types: EB—enclosed broiler, EL—enclosed layer, BR—breeder, TU—turkey, and LFR—layer free-range.

The ADKAR^®^ model revealed relatively low scores (≤3) for one or more elements in 5 out of 13 (38.5%) participating farmers during the first visit (Figure 4). Eight farmers (61.5%) scored 4 on each of the first four elements. This included two enclosed broiler, one enclosed layer, two breeder, two turkey, and one free-range layer farmer. None of the farmers scored ≤3 for all four elements. Knowledge scores differed significantly (*p* = 0.004) by education level.

### 3.4. Uptake of Actions

Each coaching session lasted for, on average, one hour. The proposed actions were mostly generic, while some were specific to a certain problem (e.g., heat treatment for mite control). Collectively, the action plans consisted of 49 different measures pertaining to the specific area of biosecurity, and one or more were agreed upon by 12 participating farmers (Table 2).

Table 3 contains an extensive list of suggested improvements. Each farm adopted, on average (±Std. dev), 4.1 ± 1.7 measures (median = 4; range = 2–8). Meanwhile, for one breeder farm (farm 10), it was not possible to reach an agreement on any actions. When the farmers were coached to make a one-time alteration (e.g., replenishment of foot baths or placing a warning sign), they invariably followed through. At the end of the study, 36 actions were recorded as adopted. Thirteen were not implemented due to the impracticality of the proposed measures or facility reconstruction, which constitutes a significant cost burden. Among them, the farmers expressed interest in five actions but generally not in the near future.

Among the external biosecurity measures, the uptake of actions was mostly related to site infrastructure. Eight proposals were deemed feasible in this category, and seven were subsequently adopted (87%). Every farm that was advised to repair the roof (*n* = 1), repair the floor (*n* = 1), keep other animals off the site (*n* = 4), and cover the winter garden (*n* = 1) considered implementing this advice. A high compliance percentage (100%) was noted for “purchasing policy” as well. Farms that were given guidance to purchase birds from a single known supplier (*n* = 2), reduce the frequency of chick deliveries to the farm (*n* = 1), and test replacement birds (*n* = 1) fully adopted the action plans. The advice to reduce the number of steps during depopulation (thinning) was not adopted, but the remaining actions (5 out of 6) under transport and depopulation were fully adopted, achieving high compliance. In particular, the practice of cleaning and disinfecting transport crates and containers upon their arrival was implemented in all five farms as recommended. In the “external biosecurity” category, improvement options for “site access” had the lowest compliance (56%). Out of the various improvement options suggested for this category, the installation of a disinfection bath or foot washer was the one that was widely adopted (100%).

For internal biosecurity management, action plans for “flock management” were adopted at rates ranging from 25% to 100%. The farmers who were given guidance on flock management were more inclined to adjust the stocking density (100%) and conduct necropsies on culled and dead birds (100%) rather than implementing other options such as maintaining the same age group in all houses (25%) or administering new vaccinations (75%). Action plans in the category “cleaning and disinfection” had the lowest compliance rates (38%). Eight action plans drawn for cleaning and disinfection were deemed feasible; however, only three were adopted, while two were planned for the future (*n* = 2). Only one action plan each was drawn up for “egg transport” and “egg management;” hence, there is no breakdown of the implementation of specific actions. Although action plans were somewhat limited here, a high compliance percentage (100%) was achieved. 

### 3.5. Biosecurity Adoption

A 6-month duration was set between the second (coaching) and third (final assessment) visits to allow for the farmers to adopt any suggested changes in biosecurity. However, in reality, the duration varied between 3.5 to 12.5 months (with a median time of 6.2 months) as a result of delays in recruiting the participants and problems in reaching out to farmers for the subsequent visits. In general, the biosecurity levels improved except for farm 10 (the breeder farm), where no action plans were drawn up (Table 4 and Table 5).

### 3.6. Attitude Change

As a score of 3 or lower on any ADKAR component signifies an obstacle to the change process [21,31], farmers were grouped into two groups (low or high) based on their ADKAR^®^ element scores, with scores of 1, 2, or 3 considered low and scores of 4 or 5 considered high (Table 6). Farmers with initial high scores remained the same (*n* = 8). Despite coaching, for two farmers, the attitude scores remained low (Figure 4). With the exception of these two farmers (farms 1 and 9) who had consistently low scores, changes in attitude were noted for the remaining three farms in the low scoring group. The fifth element, reinforcement (R), which was assessed during the final evaluation, had an average (±Std. dev) score of 3.8 (±0.4) with a median of 4. For the two farms with consistently low ADKA scores post-coaching, the “R” values were also low (=3) (Table 4). 

## 4. Discussion

The study investigated the influence of coaching on the attitudes of Belgian poultry producers towards biosecurity and the uptake of preventive measures. To the best of our knowledge, this is the first prospective intervention study to examine this in different production systems like broiler, enclosed layer, turkey, breeder, and free range. 

Coaching was used to help farmers independently set goals and find long-term solutions to a specific problem [13,14,34]. Traditional training programs or government audits may ensure compliance but have a tendency towards short-term effectiveness, according to past studies [9,11,35]. Since it has been described that farmers trust their veterinarians [36,37] and are more inclined to change if veterinarians advise them [38,39,40], the coaching in this study was performed by a veterinarian with biosecurity and poultry expertise alongside the farm veterinarian [41,42,43] because expert trainers or coaches are necessary for the correct application of biosecurity practices [44,45]. All farmers were profiled by the same coach by following the guidelines outlined by Amalraj et al. [21] to minimize observer bias.

As the “one-size-fits-all” method is neither appropriate nor effective [46], the farm team needed to understand each farm’s needs to create a farm-specific action plan. Researchers in the past [15,47,48] have already demonstrated that change occurs when the advice is tailored to farmers’ individual contexts and characteristics rather than being generic. To achieve this, face-to-face interviews and Biocheck.UGent^TM^ audits were performed to obtain firsthand information on biosecurity measures, minimizing the response bias [49] caused by farmers’ social desirability [50]. Past studies [13,22] have used similar tools to coach broiler producers, improve biosecurity, and reduce antimicrobial use. 

Yet to achieve change, it is not sufficient to understand what needs to be done; it is also crucial to know what motivates or blocks change [51]. Understanding this allows the coach to plan more successful interventions. In the current study, five poultry producers had low ADKAR^®^ scores for one or more attitude elements (Figure 4), suggesting a pre-existing barrier to change [31]. When a farmer (e.g., Farm No. 6) lacked intent to change (awareness score ≤ 3), the coach raised awareness by pointing out the consequences arising from poor biosecurity. The majority of the farmers (12/13) in this study recognized the benefits of biosecurity and, therefore, needed no further motivation on that aspect. However, awareness alone does not always influence behavior, but comprehension leads to action, according to Pike [52]. As Szulanski [53] stated, both “motivational barriers” and “knowledge barriers” have an impact on the learning and transformation processes of farmers. For farmers who want to change but lack the required knowledge (awareness and desire score > 3; knowledge score ≤ 3), guidance on how to proceed further was given (e.g., Farm No. 1). Contrary to prior studies, where lack of knowledge was mentioned as a reason for non-compliance with biosecurity [54,55], a substantial amount of knowledge (mean “K” score = 3.7 ± 0.6) about biosecurity practices existed among the farmers in this study. This was also translated into an already good implementation of numerous biosecurity measures. Yet, at the same time, there was a need for improvement in one or more areas on each farm.

Through coaching, new information was discussed alongside the farms’ biosecurity reports to expand upon existing knowledge and relate it to their farming context, as farmers are likely to adopt ideas when they can connect evidence-based concepts to their farming practices [56]. The suggested actions consisted of either a one-time change (i.e., placing a warning sign), a periodic practice (i.e., water analysis), or a habitual change in behavior (i.e., washing hands). The majority expressed interest in looking into adopting the proposed actions, although one farmer was determined not to accept any. Sometimes, disagreements arose due to differing priorities among those involved, but in such cases, the focus was on improvements where there was consensus. Forty-nine potential improvements were suggested, indicating the possibility of implementing diversity measures. The number and extent of specific biosecurity recommendations during coaching varied across farms. The achieved level of adherence to the advice (73%) was relatively high. One possible explanation is that the majority of farmers achieved high scores (>4) on the ADKA elements (Figure 4), indicating fewer barriers and, therefore, a greater acceptance of the action plan drawn during coaching. Yet the fact that it was not 100% indicates that not all changes can be achieved at once, underlying the idea that adopting biosecurity is a long-term engagement [9,35]. It is interesting to note that the biosecurity procedures that the farmers thought were most feasible and that were effectively implemented were simpler measures that were more practical and easy to execute. Examples include installing a disinfection bath or using gloves for dirty work. While some of the improvements were more straightforward and affordable, others were more complex and expensive, like heat treatment or fencing. Occasional failure to comply was due to “lack of time,” as previously found [33,35,57]. Some admitted they became demotivated by recurring problems (e.g., Salmonella persistence) despite investments. 

In all but one of farms, the biosecurity scores improved; however, farms 5 and 9 had minimal changes in the biosecurity scores despite implementing four actions on each farm (Table 4). This was due to the fact that the suggested improvements were not fully captured through the Biocheck.UGent^TM^ scoring tool. This illustrates that the tool can be used as a support to identify areas for improvement, yet one should not restrict himself/herself to the components of the scoring tool for advice on improvements. The scoring tool, along with the knowledge and expertise of the parties involved (farmer, farm veterinarian, and coach), is what ultimately yields a list of possible improvements. Nevertheless, farmers found Biocheck.UGent^TM^ helpful in reviewing their biosecurity practices, raising awareness of the importance of good biosecurity, and identifying potential weaknesses that were previously overlooked. 

According to Olsen et al. [58] and Mankad [59], behavioral change, especially concerning risk-reduction practices, can be a difficult long-term process in which motivation is high merely at the beginning and the real challenge lies in sustaining these behaviors over time [50]. The reinforcement score (median = 4) indicates that the change did have a positive impact on farmers. This element is persuasive about the need for biosecurity, as it represents an effort to measure biosecurity impact [33] and indicates a likelihood of persistence in change adoption [13,20,21]. While sustainability depends on the exact biosecurity measures employed [60], due to the study’s short duration, long-term sustainability could not be investigated. The authors advise exercising caution and recommend longer observation periods before interpreting the reinforcement score. 

The success of this study is rooted in the facilitation of communication between the coach, veterinarian, and farmer regarding biosecurity, which has fostered comprehension and potential consensus on diverse perspectives [61]. However, when interpreting the above results, some study limitations should be taken into account. Visit 1 served as the control data due to the lack of parallel control farms, making each farm as its own control the most feasible approach in this study. Such an approach has been used in the past [10,13,62]. Given the time-consuming nature of on-farm interviews and interventions [63], it is understandable that this project permitted only a limited number of study farms. A larger, more diversified sample of farmers may help us understand the learning differences [4]. A recent study has shown the success of coaching in enhancing biosecurity compliance in Italian poultry farms [33] on a somewhat larger scale than ours, which demonstrates the broad scope of this approach. Within the framework of this project, a study conducted by de Carvalho Ferreira et al. [64] has shown that providing personalized coaching on the farm demanded a greater amount of time and incurred higher expenses for the facilitator (i.e., the coach). This economic study of similar interventions may provide additional motivation. The farm selection process, based on farmers’ willingness rather than random sampling, leads to potential selection bias. Another concern is the potential for undetected issues to go unaddressed. For instance, action plans may not include rodent management if auditors found no rats or the farmer did not raise concern. Repeated visits and coaching over a longer period of time can help overcome such issues and achieve sustainable and broad improvement while also building credibility and trust [65].

## 5. Conclusions

The incomplete implementation of biosecurity measures by farmers is a complex issue, with inconsistent support likely being a key factor. Considering sociological obstacles, using the ADKAR^®^ model during coaching supported by biosecurity quantification with a scoring tool like Biocheck.UGent^TM^ can offer a useful foundation for structuring veterinarian–farmer discussions on farm biosecurity strategies. A significant rise in farm biosecurity levels, as demonstrated by the Biocheck.UGent^TM^ survey results, suggests that by providing specific guidance to enhance hygiene procedures, it is possible to overturn existing practices. A shift in mindset (barrier scores) should also be regarded as one of the key success factors of this study. 

## Figures and Tables

**Table 1:** Results from the variables used in the analysis of the characteristics of the farmers and comparison of attitude and biosecurity scores according to different subgroups.

Demographic Variables	N=	%	Awareness Score	Desire Score	Knowledge Score	Ability Score	External Biosecurity Score	Internal Biosecurity Score	Total Biosecurity Score
			$\bar{x}$ ± SD	$\bar{x}$ ± SD	$\bar{x}$ ± SD	$\bar{x}$ ± SD	$\bar{x}$ ± SD	$\bar{x}$ ± SD	$\bar{x}$ ± SD
**Age group**									
<35 years old	3	23.1	4.0 ± 0.0 ^a^	4.0 ± 0.0 ^a^	3.7 ± 0.6 ^a^	4.0 ± 0.0 ^a^	64.3 ± 4.2 ^a^	67.0 ± 14.0 ^a^	65.0 ± 2.6 ^a^
35–50 years old	3	23.1	4.0 ± 0.0 ^a^	4.0 ± 0.0 ^a^	4.0 ± 0.0 ^a^	3.7 ± 0.6 ^a^	64.3 ± 2.1 ^a^	81.3 ± 6.7 ^a^	69.3 ± 2.3 ^a^
>50 years old	7	53.8	3.9 ± 0.4 ^a^	3.9 ± 0.4 ^a^	3.6 ± 0.8 ^a^	3.9 ± 0.4 ^a^	67.3 ± 9.4 ^a^	67.4 ± 8.4 ^a^	67.0 ± 7.5 ^a^
**Gender**									
Male	10	76.9	3.9 ± 0.3 ^a^	3.9 ± 0.3 ^a^	3.6 ± 0.7 ^a^	3.8 ± 0.4 ^a^	64.9 ± 7.2 ^a^	68.4 ± 9.8 ^a^	65.9 ± 6.1 ^a^
Female	3	23.1	4.0 ± 0.0 ^a^	4.0 ± 0.0 ^a^	4.0 ± 0.0 ^a^	4.0 ± 0.0 ^a^	69.3 ± 6.7 ^a^	77.7± 12.1 ^a^	71.0 ± 1.0 ^a^
**Education**									
Primary school	1	7.7	4.0 ± 0.0 ^a^	4.0 ± 0.0 ^a^	4.0 ± 0.0 ^a^	4.0 ± 0.0 ^a^	56.0 ± 00 ^a^	58.0 ± 00 ^a^	57.0 ± 00 ^a^
Secondary school	11	84.6	3.9 ± 0.3 ^a^	3.9 ± 0.3 ^a^	3.8 ± 0.4 ^b^	3.8 ± 0.4 ^a^	65.8 ± 6.2 ^a^	72.3 ± 10.6 ^a^	67.6 ± 5.2 ^a^
University	1	7.7	4.0 ± 0.0 ^a^	4.0 ± 0.0 ^a^	4.0 ± 0.0 ^a^	4.0 ± 0.0 ^a^	77.0 ± 00 ^a^	64.0 ± 00 ^a^	71.0 ± 00 ^a^
**Production type**									
Enclosed broiler	4	30.8	4.0 ± 0.0 ^a^	4.0 ± 0.0 ^a^	3.3 ± 1.0 ^a^	4.0 ± 0.0 ^a^	64.3 ± 7.0 ^a^	61.8 ± 7.5 ^a^	63.5 ± 5.3 ^a^
Enclosed layer	2	15.4	3.5 ± 0.7 ^a^	4.0 ± 0.0 ^a^	4.0 ± 0.0 ^a^	4.0 ± 0.0 ^a^	67.5 ± 13.4 ^a^	61.0 ± 4.2 ^a^	64.5 ± 9.2 ^a^
Breeder	4	30.8	4.0 ± 0.0 ^a^	3.8 ± 0.5 ^a^	3.8 ± 0.5 ^a^	3.5 ± 0.6 ^a^	67.3 ± 9.2 ^a^	76.3 ± 6.7 ^a,b^	69.8 ± 5.7 ^a^
Turkey	2	15.4	4.0 ± 0.0 ^a^	4.0 ± 0.0 ^a^	4.0 ± 0.0 ^a^	4.0 ± 0.0 ^a^	65.0 ± 0.0 ^a^	81.0 ± 5.7 ^a,b^	69.0 ± 1.4 ^a^
Free-range layer	1	7.7	4.0 ± 0.0 ^a^	4.0 ± 0.0 ^a^	4.0 ± 0.0 ^a^	4.0 ± 0.0 ^a^	66.0 ± 0.0 ^a^	87.0 ± 0.0 ^b^	72.0 ± 0.0 ^a^

^a,b^ For each variable, values in the same column not sharing the same superscript are significantly different at *p* < 0.05.

**Table 2:** Action plans (AP) adopted by the farm team following coaching.

	No.	%
No adoption of any AP in the farm	1	7.7
Adoption of minimum one AP in the farm	12	92.3
	13	100

**Table 3:** Breakdown of the action plans by measure and the percentage of measures implemented by farmers.

Categories for Improvement	N=	List of Action Plans *	Number of Farms (%)		
			Agreed ^a^	Adopted ^b^	Planning for Future
Site infrastructure	1	Protect farm site with fences	3 (23)	1 (33)	
	2	Cement floor to improve drainage	1 (8)	1 (100)	
	3	Improve rodent control	3 (23)	1 (33)	1 (33)
	4	Avoid other livestock/pets on site	4 (31)	2 (50)	
	5	Avoid other poultry (hobby birds also) on site	2 (15)	1 (50)	
	6	Roof repair	1 (8)	1 (100)	
	7	Cover winter garden	1 (8)	1 (100)	
	8	Close ventilation outlet	1 (8)	0	
Site entrance	1	Park car away from poultry house	2 (15)	0	2 (100)
	2	Distinction between clean and dirty road	2 (15)	0	
	3	Minimize/control visitors	3 (23)	1 (33)	
	4	No entry for drivers/suppliers into houses	3 (23)	1 (33)	
	5	Warning signs: “STOP”	2 (15)	1 (50)	
	6	Accessibility only via hygiene lock for visitors/staff	2 (15)	0	
	7	Farm hygiene lock installation	2 (15)	0	
	8	Disinfection bath/foot washer installation	4 (31)	4 (100)	
	9	Regular replenishment of disinfection bath	2 (15)	2 (100)	
Staff/visitors	1	Personnel limited to one specific farm	5 (38)	1 (20)	
	2	Hand washing/hand sanitizer used	2 (15)	1 (50)	
	3	Gloves for dirty work (e.g., carcass collection)	2 (15)	2 (100)	
	4	Clean overalls and boots for visitors/staff	3 (23)	1 (33)	
Purchase policy	1	Buying from same supplier	2 (15)	2 (100)	
	2	Reduce the chick delivery frequency	1 (8)	1 (100)	
	3	Replacement males to be tested at spiking	1 (8)	1 (100)	
Transport and depopulation	1	Reduce depopulation steps	2 (15)	0	
	2	Clean and disinfect crates and containers upon arrival	5 (38)	5 (100)	
	3	Clean and disinfect shared catching machine	1 (8)	1 (100)	
	4	Clean overalls and boots for catching crew	1 (8)	1 (100)	
	5	Clean and disinfect loading area	1 (8)	1 (100)	
	6	Clean and disinfect egg trucks upon arrival ^c^	2 (15)	2 (100)	
Feed and water supplies	1	Clean feed spillage immediately	1 (8)	1 (100)	
	2	Water analysis	2 (15)	1 (50)	
	3	Water treatment	2 (15)	0	
Flock management	1	Reduce stocking density	2 (15)	2 (100)	
	2	Maintain same age group in all houses	4 (31)	1 (25)	1 (25)
	3	New vaccination	2 (15)	1 (50)	
	4	Necropsy of culled and dead birds	1 (8)	1 (100)	
Cleaning and disinfection	1	Design a protocol with vets	2 (15)	0	
	2	Use detergent during wet cleaning	1 (8)	1 (100)	
	3	Clean and disinfect of litter spreading machines	1 (8)	0	
	4	Heat treatment for mite control	3 (23)	3 (100)	
	5	Disinfect egg room after every collection ^d^	2 (15)	0	1 (50)
	6	Clean and disinfect silo	3 (23)	0	1 (33)
	7	Clean and disinfect waterlines	1 (8)	0	
	8	Clean and disinfect materials (buckets and equipment)	1 (8)	1 (100)	
Measures between houses	1	Use recognizable, separate, color-coded materials	3 (23)	1 (33)	
	2	House hygiene lock installation	3 (23)	2 (67)	
	3	Use clean house-specific overalls and boots	1 (8)	0	
	4	Disinfection bath/mat installation	1 (8)	1 (100)	
TOTAL			49	36	5

N = number of included measures per category. ^a^ The column “agreed” shows on what percentage of the farms the action was agreed upon during visit 2. ^b^ The column “adopted” shows on what percentage of the farms the provided action was actually performed (based on observations and evaluations during visit 3).^c^^,d^ Action not offered for meat poultry production (broiler and turkey). * The number of recommended options (*n* = 49) exceeded the number of participating farms due to the decision made by the coach, herd veterinarian, and farmer to formulate the action plan based on Biocheck.UGent^TM^ scores and the observed weaknesses.

**Table 4:** Changes in biosecurity and attitude following coaching.

Farm	Production Type	Validation Phase Days (Months)	Adopted Actions *n*=	Total Biosecurity Score (%) before Coaching	Total Biosecurity Score (%) after Coaching	Change in Biosecurity Score (+/− Points)	Change in ADKA Score	Reinforcement Score
1	EB	152 (4.9)	4	57	60	+3	No ***	3
2	EB	223 (7.3)	2	64	68	+4	No *	4
3	EB	108 (3.5)	3	70	75	+5	No *	4
4	EB	122 (4.0)	3	63	68	+5	Yes **	4
5	EL	191 (6.2)	4	71	72	+1	No *	4
6	EL	382 (12.5)	8	58	65	+7	Yes **	3
7	BR	196 (6.4)	5	68	71	+3	Yes **	4
8	BR	189 (6.2)	2	68	73	+5	No *	4
9	BR	138 (4.5)	4	65	66	+1	No ***	3
10	BR	209 (6.9)	0	78	78	-	No *	4
11	TU	136 (4.7)	6	70	72	+2	No *	4
12	TU	243 (7.9)	4	68	71	+3	No *	4
13	LFR	142 (4.6)	4	72	75	+3	No *	4

Production type: EB—enclosed broiler, EL—enclosed layer, BR—breeder, TU—turkey, and LFR—layer free-range. * High ADKA ranking (score = 4) for all four elements on visit 1. ** Improvement in ADKA element (s) (see Figure 4). *** Scores of one or more elements remained low following coaching.

**Table 5:** Mean ± Std. deviation (SD), median (range), and change in Biocheck.UGent^TM^ scores between pre- and post-coaching assessments after implementation of an on-farm individual coaching program for 13 farmers in biosecurity compliance in poultry production. Significant *p*-values for the paired samples *t*-test are presented.

Main Categories	Before Coaching					After Coaching					*p*-Value
	Mean	SD	Median	Min	Max	Mean	SD	Median	Min	Max	
External biosecurity (EB)	65.9	7.1	65	56	81	69.1	6.2	69	58	81	0.003
Internal biosecurity (IB)	70.5	10.6	70	53	87	73.8	8.4	74	63	87	0.012
Total biosecurity (TB)	67.1	5.7	68	57	78	70.3	5.7	71	60	78	0.002

**Table 6:** The number of poultry farms are grouped according to their score (low/high) for each ADKAR^®^ element. The ADKAR^®^ elements were divided into low (score 1, 2, or 3) and high (score 4 or 5) groups.

		Awareness		Desire		Knowledge		Ability		Reinforcement	
		LOW	HIGH	LOW	HIGH	LOW	HIGH	LOW	HIGH	LOW	HIGH
**Poultry Farmers** **(*n* = 13)**	Before coaching	1	12	1	12	3	10	2	11	-	-
	After coaching	0	13	1	12	2	11	1	12	3	10

## Data Availability

The data presented in this study are available upon request from the corresponding author. The data are not publicly available due to privacy and confidentiality agreements with the participants.

## Supplementary Materials

The following supporting information can be downloaded at: https://www.mdpi.com/article/10.3390/ani14172498/s1.
