# Supplementary material for: Assessing the Impact of On-Farm Biosecurity Coaching on Farmer Perception and Farm Biosecurity Status in Belgian Poultry Production
Source: Animals (Basel). 2024 Aug 28;14(17):2498. doi: 10.3390/ani14172498 (PMC11394560; doi:10.3390/ani14172498)
Supplement: Supplementary file 1 [file animals-14-02498-s001.zip › animals-3156863-supplementary.pdf]

FACULTY OF VETERINARY MEDICINE
accredited by EAEVE

biocheck .ugent

BIOCHECK POULTRY

Breeders

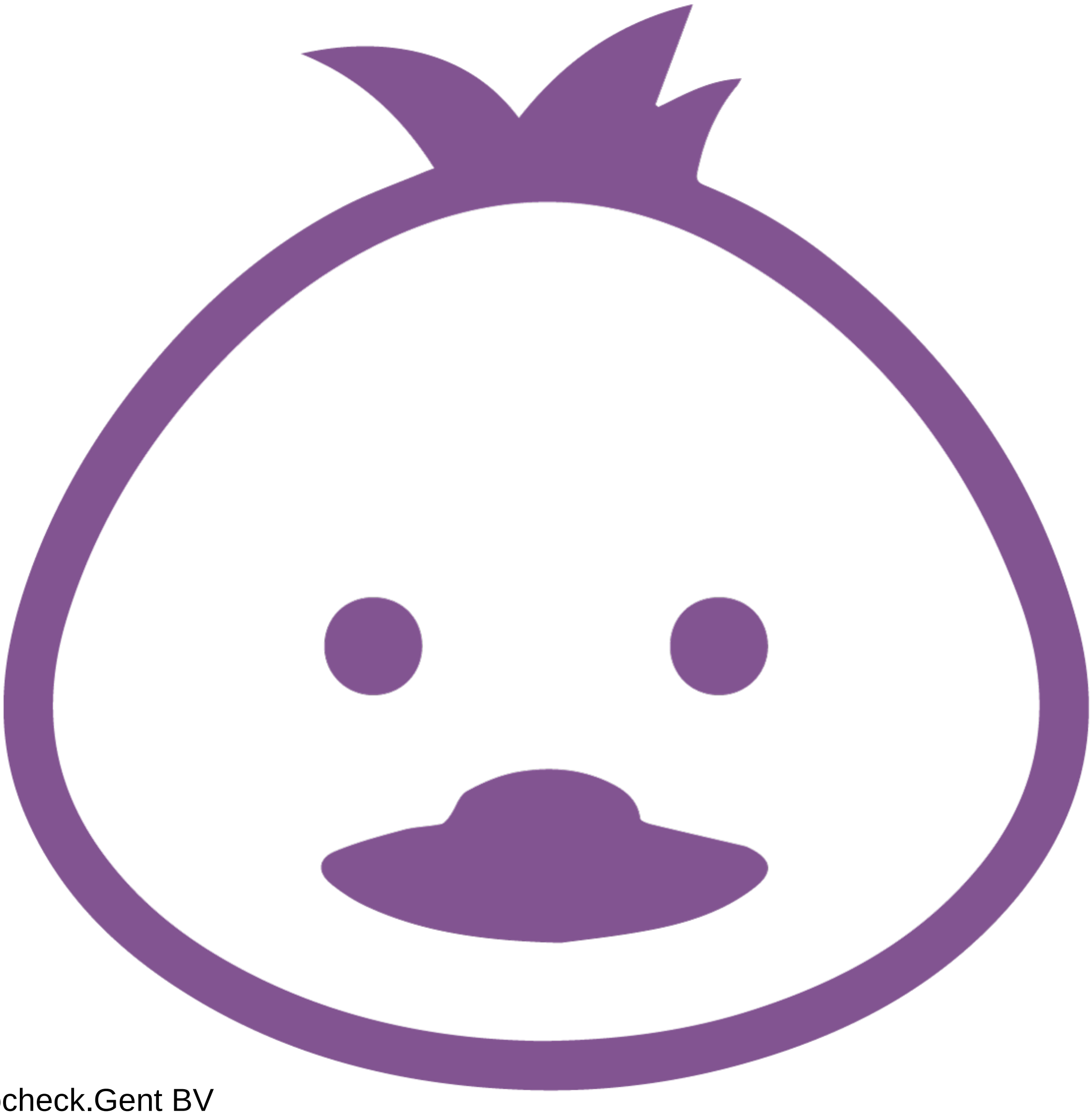

Biocheck.Gent BV

E: info@biocheckgent.com
T: 0032 92 64 75 43

www.biocheckgent.com

GHENT UNIVERSITY

~. Farm characteristics

I. How many breeders are there on the farm?

....

II. How many years of experience in keeping breeders does the person in charge have?

....

III. How many people are working on the breeder farm?

....

IV. Which breeder species do you keep on your farm?

Check any that apply.
□ Broiler breeders
□ Laying hen breeders
□ Turkey breeders
□ Duck breeders
□ Guinea fowl breeders
□ Others

V. What type of management system is used on the farm?

Select one option.
○ All-in/All-out
○ Mixed
○ Multiple ages

VI. What type of housing system is used on the farm?

Check any that apply.
□ Cages
□ Aviary
□ Floor system
□ Others

VII. How old (in years) is the oldest building in which birds are being kept?

....

VIII. How old (in years) is the newest building in which birds are being kept?

....

A. Infrastructure, location and housing

1. Is the outside of the animal facilities, especially the working area (around the front walls) in concrete/paved and is it clean (e.g. removal of weeds, waste, …)? *(required)*

Select one option.
○ Yes, it's completely paved and clean
○ It's only partially paved and clean
○ No

2. Is the farm fenced to prevent free entry of visitors/ animals into the premises? *(required)*

Select one option.
○ Yes, it's completely fenced
○ It's only partially fenced
○ No

3. Is there stagnant or running water within a 1-kilometre radius (0.6 miles) of the farm? *(required)*

Select one option.
○ No
○ Yes

4. At what distance (straight-line) is the nearest neighbouring poultry farm/ slaughterhouse/ rendering company located? *(required)*

Select one option.
○ More than 1 Km (0.6 miles)
○ Between 500m - 1 Km (between 0.3 and 0.6 miles)
○ Less than 500m (Less than 0.3 miles)

5. Is manure from other poultry farms spread on the neighbouring farmlands (within a 500-metre (0.3 miles) radius)? *(required)*

Select one option.
○ Never
○ Sometimes
○ Often

6. Does poultry transport frequently occur (i.e. minimum once a day) via the public road (road less than 100 metres (328 feet) from your farm) where your farm is located at (e.g. due to the location of a slaughterhouse in the neighbourhood…)? *(required)*

Select one option.
○ No
○ Yes

7a. Are any other farm animals being kept on the same farm site? *(required)*

Select one option.
○ No
○ Yes
If "No" is chosen, go to question 8a.

7b. Check any that apply. *(required)*

Check any that apply.
□ Pigs
□ Horses
□ Cattle
□ Sheep
□ Goats
□ Others

8a. Are any poultry species other than breeders being kept on the same farm site? *(required)*

Select one option.
○ No
○ Yes
If "No" is chosen, go to question 9.

8b. Check any that apply. *(required)*

Check any that apply.
□ Broilers
□ Laying hens
□ Turkeys/ ducks/ geese
□ Guinea Fowls/ quails
□ Non-commercial poultry/ backyard
□ Pet birds/ others

9. Do other farm animals/ poultry/ pets that are being kept on the same farm site have access to the breeder houses? *(required)*

Select one option.
○ No
○ Yes

10a. Which wild birds do you see on the farm premises? *(required)*

Select one option.
○ None
○ Migratory birds
○ Residential/ indigenous/ local birds
If "None" is chosen, go to question 11.

10b. Check any that apply. *(required)*

Check any that apply.
□ Shorebirds (gulls and related)
□ Waterfowl
□ Raptor predators
□ Corvids (crows and related, including magpies)
□ Heron, storks, cranes
□ Pigeons and related
□ Small resident birds (e.g. starlings)

11. Is a rodent control program present on the farm (other than cats)? *(required)*

Select one option.
○ Yes, a professional pest control company has been hired periodically
○ Yes, farmer has established his own pest control programme periodically
○ Yes, pest control is performed only if infestation is noticed (via rodent trap)
○ No pest control

12. Are bird- and vermin-proof grids placed on the air inlets? *(required)*

Select one option.
○ Yes
○ No

B. Organization of the farm and supply of materials

13. Does the farm follow a written biosecurity plan? *(required)*

Select one option.
○ Yes
○ No

14. Have the farmer and farmworkers received formal training on biosecurity? *(required)*

Trainings can be diploma, certified e-learning courses, training from regulatory organizations, workshops, webinars.

Select one option.
○ Yes, both have received training on biosecurity
○ Yes, but only one of them has received training on biosecurity
○ No, neither of them have received training on biosecurity

15. Does the farm have entry restriction for non-essential vehicles for which there is parking space outside the farm premises? *(required)*

Essential vehicles: poultry delivery and transport trucks, feed delivery trucks, egg transport truck.
Non-essential vehicles: vehicle of vets, visitors, farm personnel.

Select one option.
○ Yes
○ No

16. Is there a clear separation between the clean and the dirty area of the farm premises? *(required)*

The clean road/area is the area of the production site with restricted access, i.e. this is the area where only animals from the farm, persons after they have applied the hygienic measures in the hygiene lock, and farm-specific materials and vehicles are allowed. The dirty area comprises all other parts of the farm where visitors, external vehicles, … have access to. The dirty area also includes the carcass storage facility.

Select one option.
○ Yes
○ No

17. Are the wheels of vehicles (including feed truck, egg transport truck, poultry transport truck, etc) always disinfected when entering the farm (e.g. driving through disinfection baths / spray system / etc..)? *(required)*

Select one option.
○ Always
○ Sometimes
○ Never

18. Is there any material/ equipment being shared with other farms that enters the breeder houses and/or has contact with your breeders (e.g. bird catching machines)? *(required)*

Select one option.
○ No
○ Yes

19. Are specific measures taken for the introduction of material/ equipment (e.g. UV-disinfection unit, alcohol disinfection, soap and water)? *(required)*

Select one option.
○ Yes
○ No

20. Is fresh litter used for every new batch? *(required)*

Select one option.
○ Yes
○ No
If "No" is chosen, go to question 23.

21. Is the quality of the litter (for bedding) checked at placement? *(required)*

Select one option.
○ Yes
○ I don't know
○ No

22. Is the stored replacement litter at all times protected from water/bird/vermin? *(required)*

Select one option.
○ Yes
○ Replacement litter is not stored
○ No

C. Visitors and personnel

23. Are visitors obliged to notify their presence before entering the breeder farm (e.g. visitor's register)? *(required)*

Select one option.
○ Yes
○ No

24. Is a non-bird contact period (longer than 12 hours) expected of all visitors before they are allowed to enter the breeder houses? *(required)*

Select one option.
○ Yes
○ No

25. Is a FARM hygiene lock present (when entering the farm)? *(required)*

Select one option.
○ Yes
○ No
If "No" is chosen, go to question 28

26. Are the breeder houses accessible for visitors and personnel only through the FARM hygiene lock? *(required)*

Select one option.
○ Yes
○ No

27. Is there a strict separation between the clean and dirty area of the FARM hygiene lock? *(required)*

Strict separation: by wooden plank/ bench or marking with paint/ tape.

Select one option.
○ Yes
○ No

28. Is there a shower facility and is it always used by visitors/ personnel before they enter the breeder farm? *(required)*

Select one option.
○ Yes
○ Only for visitors, not for personnel
○ No shower facility
○ Present but not used
If "Yes" is chosen, go to question 30.

29. Do visitors/ personnel practice hand washing or hand disinfection before they enter the breeder farm? *(required)*

Select one option.
○ Yes
○ No

30. Are farm-specific clothes always used by visitors/ personnel before they are allowed to enter the breeder farm? *(required)*

Select one option.
○ Yes
○ No

31. Are farm-specific shoes always used by visitors/ personnel before they are allowed to enter the breeder farm? *(required)*

Select one option.
○ Yes
○ No

32. How many times per year is access to the breeder houses granted to non-essential visitors? *(required)*

Essential visitors: veterinarian, representatives of the feed companies, vermin control.

Select one option.
○ Access is never granted
○ Access is granted, but less than 12 times a year
○ Access is granted more than 12 times a year

33. Do all farmworkers (including the farm owner) abide by the access rules? *(required)*

Select one option.
○ Always
○ Sometimes
○ Never

34. Does the farmer or farmworkers also keep any other poultry at home or have a bird-related hobby (e.g. bird hunting, pigeon breeding, cock fighting)? *(required)*

Select one option.
○ No
○ Yes

35. Are farm and hatchery employees shared? *(required)*

Select one option.
○ No hatchery on the premises
○ Never
○ Sometimes
○ Always

D. Purchase of adult breeders

36. Are the breeders (during the last 2 years of farming) always bought from the same supplier? *(required)*

Select one option.
○ Always the same supplier
○ Sometimes the same supplier
○ Never the same supplier

37. Whenever breeders are bought from another farm, is proof requested to ensure that the sanitary statute and health management of the farm of origin is equal or higher than your own farm? *(required)*

Select one option.
○ Yes
○ No

38. Are the bought breeders always first delivered to your farm, i.e. before other farms are supplied by the same transport vehicle? *(required)*

Select one option.
○ Always
○ Sometimes
○ Never
○ I don't know

39. Is it verified if the transport vehicles (including the transport crates and containers) are cleaned and disinfected before the birds are loaded at the suppliers company? *(required)*

Select one option.
○ Always
○ Sometimes
○ Never
○ I don't know

40. What happens with the breeders after their first production cycle? *(required)*

Select one option.
○ The breeders are always slaughtered
○ The breeders are sometimes kept for a second production period after moulting
○ The breeders are always kept for a second production period after moulting

41. Are replacement males added to the flocks (around week 40 – 45) to compensate for the decline in fertility (= spiking)? *(required)*

Select one option.
○ No
○ Yes, addition of young breeder males (from another farm) to the older flocks
○ Yes, switching of breeder males between different poultry houses on the same farm
If "No" or "Yes, switching of breeder males between different poultry houses on the same farm" is chosen, go to question 45.

42. Do the younger breeder males (during the last 2 years) always come from the same supplier? *(required)*

Select one option.
○ Always the same supplier for all the poultry houses at the farm
○ Always the same supplier for every separated poultry house at the farm (e.g. House 1 has supplier A and House 2 has supplier B…)
○ Mostly different suppliers

43. Are the young breeder males serological tested before being introduced to the flock? *(required)*

Select one option.
○ Yes
○ No

44. Are replacement males being kept separately in quarantine before introduction? *(required)*

Select one option.
○ Always
○ Sometimes
○ Never

E. Depopulation of breeders

45. Are the transport vehicles (including the transport crates and containers) for breeders empty on arrival at the farm? *(required)*

Select one option.
○ Always
○ Sometimes
○ Never
If "Sometimes" or "Never" is chosen, go to question 47.

46. Are the transport vehicles (including transport crates and container) always cleaned and disinfected before arrival at the farm? *(required)*

Select one option.
○ Always
○ Sometimes
○ Never

47. Are the empty transport crates and containers for breeders always cleaned and disinfected on arrival at the farm? *(required)*

Select one option.
○ Always
○ Sometimes
○ Never

48. Are the transport crates and containers transported in- and outside the breeder houses with farm-specific equipment (e.g. loader)? *(required)*

Select one option.
○ Yes
○ No, not relevant as crates don't enter the stables
○ No, there is no farm-specific equipment

49. Are the loading and unloading areas cleaned and disinfected after each depopulation? *(required)*

Select one option.
○ Yes, cleaned and disinfected
○ Only cleaned
○ Not cleaned and disinfected

50. In how many steps does the depopulation of a breeder house take place? *(required)*

Select one option.
○ In one step
○ In two steps
○ In more than two steps

F. Transport of eggs

51. Are the eggs that are ready for transport stored in a specific store room (i.e in a room different from the egg room)? *(required)*

Select one option.
○ Yes
○ No

52. Is the transport vehicle for the eggs empty on arrival at the farm? *(required)*

Select one option.
○ Always
○ Sometimes
○ Never
If "Sometimes" or "Never" is chosen, go to question 54.

53. Is the transport vehicle for eggs cleaned and disinfected before arrival at the farm? *(required)*

Select one option.
○ Always
○ Sometimes
○ Never

54. Does the driver have access to the egg facilities of the farm? *(required)*

Select one option.
○ No, the driver doesn't have access at all
○ Yes, but only to the specific storeroom
○ Yes, but only to the egg room
○ Yes, the driver has access to both the egg room and specific storeroom

55. Are eggs being sold at the farm? *(required)*

Select one option.
○ No
○ Yes
If "No" is chosen, go to question 57.

56. Do people enter the farm to buy eggs? *(required)*

Select one option.
○ No. The eggs can be bought without entering the farm (e.g. from the public road)
○ Yes, they enter the farm; but they stay outside the egg room or specific storeroom
○ Yes, they enter the farm and they go inside the egg room and/or specific storeroom

G. Feed and water supply

57. Can the feeding company fill up the silos/deliver feed without entering the clean area? *(required)*

Select one option.
○ Yes
○ Partially
○ No

58. Does the feed supplier have access to the houses where direct contact with the birds is possible? *(required)*

Select one option.
○ Never
○ Sometimes
○ Always

59. Are the feed silos or the feed storage rooms (storage of complete feed or concentrate) completely sealed against water, birds and vermin? *(required)*

Select one option.
○ Yes
○ No

60. How often a year does the feeding company fill up the silos or deliver feed? *(required)*

Select one option.
○ Less than 5 times a year
○ Between 5 and 20 times a year
○ Between 21 and 35 times a year
○ More than 35 times a year

61. Is the feed delivery truck disinfected and does the company certify this? *(required)*

Select one option.
○ Yes
○ No

62. In case of feed spillage (outside near the silo) is it immediately cleaned? *(required)*

Select one option.
○ Always
○ Sometimes
○ Never

63. Is the entire water delivery system (including a water header tank) covered at all times? *(required)*

Select one option.
○ Yes
○ No

64. How often are bacteriological analyses of the drinking water performed? *(required)*

Select one option.
○ Yearly or more frequent
○ Every two years
○ Less frequent than every two years
○ Never
If "Never" is chosen, go to question 66.

65. Where are the water samples for the bacteriological analyses taken? *(required)*

Select one option.
○ Both at the source and the last drinker
○ At the last drinker
○ At the source
○ From the tank in the house anteroom
○ Other (e.g. first drinker)

H. Manure and carcass removal

66. Is manure being stored on the farm? *(required)*

Select one option.
○ No
○ Yes
If "No" is chosen, go to question 68.

67. Is the manure stored in a fully closed container or compartment? *(required)*

Select one option.
○ Yes
○ No

68. Is the manure removed and disposed off through the dirty road? *(required)*

Select one option.
○ Yes
○ No

69. Is there a dedicated carcass storage? *(required)*

Select one option.
○ Yes
○ Not relevant, carcasses are immediately processed
○ No
If "No" or "Not relevant, carcasses are immediately processed" is chosen, go to question 73.

70. Is the carcass storage cooled? *(required)*

Select one option.
○ Yes
○ No

71. Is the carcass storage space closed to prevent vermin, dogs or cats from having access to carcasses? *(required)*

Select one option.
○ Yes, it's completely closed
○ It's only partially closed
○ No

72. Is this carcass storage space cleaned and disinfected each time after carcass collection? *(required)*

Select one option.
○ Always, after each collection
○ Sometimes after each collection
○ No, only after every production round
○ Never

73. What happens with the carcasses? *(required)*

Select one option.
○ The carcasses are composted
○ The carcasses are buried/ burned
○ The carcasses are stored and collected by a rendering company
If "The carcasses are buried/ burned" is chosen, go to question 75; If "The carcasses are stored and collected by a rendering company" is chosen, go to question 76.

74. Are the carcasses composted in a closed system? *(required)*

Select one option.
○ Yes, they are composted inside a building that can be completely closed
○ Yes, they are composted outside, enclosed with plastic
○ No
Go to question 77.

75. How are the carcasses buried/burned? *(required)*

Buried in appropriate soil: deep burial in pits away from groundwater source.

Select one option.
○ They are burned in an approved incinerator on the farm
○ They are buried in the appropriate soil on the farm
○ Other
Go to question 77.

76. Can the carcasses be collected by the rendering company without entering the farm premises (e.g. from the public road) or by driving through the dirty road? *(required)*

Select one option.
○ Yes
○ No

77. Is the material used for the removal of dead birds out of the poultry houses (e.g. buckets, wheelbarrow) cleaned and disinfected after each use? *(required)*

Select one option.
○ Always, after each use
○ Sometimes
○ No material used, carcass removed by hand
○ Never

78. Are protective measures being taken when manipulating dead birds (using gloves or washing hands)? *(required)*

Select one option.
○ Always
○ Sometimes
○ Never

I. Disease management

79. Is there a regular (i.e. at least once a year) evaluation made of the disease status of the farm (e.g. serology, trends in slaughterhouse findings, etc)? *(required)*

Select one option.
○ Yes
○ No

80. How often are the dead birds removed from the breeder house? *(required)*

Select one option.
○ Two or more times a day
○ Once a day
○ Less than once a day

81. Is the flock checked on a daily basis? *(required)*

Select one option.
○ Yes
○ No

82. Is a poultry health management programme in place, for which regular farm visits (e.g. by your veterinarian(s)) are performed? *(required)*

Select one option.
○ Yes
○ No

83. Does the farm maintain a multi-age flock? *(required)*

Select one option.
○ No, single-age flock
○ Yes, different age categories, which are sorted by poultry house
○ Yes, different age categories within one poultry house
If "No, single-age flock" or "Yes, different age categories within one poultry house" is chosen, go to question 85a.

84. Is farm work, per poultry house, performed from young to older birds (per house)? *(required)*

Select one option.
○ Always
○ Sometimes
○ Never

J. Measures between compartments

85a. Are there multiple breeder houses present on the farm? *(required)*

Select one option.
○ No
○ Single house with multiple interconnected compartments
○ Yes
If "No" or "Single house with multiple interconnected compartments" is chosen, go to question 96.

85b. How many breeder houses are there on the farm? *(required)*

....

86. How are the different houses accessed? *(required)*

Select one option.
○ The different houses are independently accessed
○ The different houses are connected by corridors
○ Both
If "The different houses are independently accessed" is chosen, go to question 89.

87. Are the central corridor, concrete walking pavements and other common places between different breeder houses cleaned after each production cycle? *(required)*

Select one option.
○ Yes
○ No

88. Are the central corridor, concrete walking pavements and other common places between different breeder houses disinfected after each production cycle? *(required)*

Select one option.
○ Yes
○ No

89. Is a HOUSE hygiene lock used by visitors/personnel before they enter the breeder houses? *(required)*

Select one option.
○ Yes, always used
○ No, HOUSE hygiene lock not used
If "No, HOUSE hygiene lock not used" is chosen, go to question 91.

90. Is there a strict (clear) separation (bench or marking with paint/ tape) between the clean and dirty area of the HOUSE hygiene lock? *(required)*

Select one option.
○ Yes
○ No

91. Do visitors/personnel practice hand washing or hand disinfection before entering the breeder house? *(required)*

Select one option.
○ Yes
○ No

92. Are HOUSE-specific clothes always used before entry into the breeder house? *(required)*

Select one option.
○ Yes
○ No

93. Are HOUSE-specific shoes always used before entry into the breeder house? *(required)*

Select one option.
○ Yes
○ No

94. Is there clearly recognizable, separate material available for each breeder house? *(required)*

Select one option.
○ Yes, separate material available for each breeder house
○ Yes, separate material available for each breeder house but these are occasionally shared
○ No

95. Does every house has its own material to store the eggs before transportation to the egg collecting room? *(required)*

Select one option.
○ Yes
○ No, because the automatic system allows eggs to go straight to the egg collection room
○ No

K. Cleaning and disinfection

96. Are the breeder houses cleaned after every production cycle? *(required)*

Select one option.
○ Yes, dry and wet cleaning is done after each cycle
○ Yes, however, only dry cleaning is done after each cycle
○ No
If "Yes, however, only dry cleaning is done after each cycle" or "No" is chosen, go to question 99.

97. Are the breeder houses soaked with water before the start of cleaning? *(required)*

Select one option.
○ Always
○ Sometimes
○ Never

98. Is detergent/soap added to the water during cleaning? *(required)*

Select one option.
○ Always
○ Sometimes
○ Never

99. Are the breeder houses disinfected after every flock? *(required)*

Select one option.
○ Yes
○ No
If "No" is chosen, go to question 101.

100. Are the breeder houses dry before starting the disinfection? *(required)*

Select one option.
○ Always
○ Sometimes
○ Never

101. Is the efficacy of cleaning and disinfection checked after each production cycle (e.g. hygienogram or swabs)? *(required)*

Select one option.
○ Always
○ Sometimes
○ Never

102. Is there a protocol for the cleaning and disinfection of material after each production cycle and is this protocol always abided by? *(required)*

Select one option.
○ Yes
○ No

103. Is the egg storage room regularly cleaned? *(required)*

Select one option.
○ Yes, after each outgoing egg transport
○ Yes, after each production cycle
○ No
If "No" is chosen, go to question 105.

104. Is the egg storage room regularly disinfected? *(required)*

Select one option.
○ Yes, after each outgoing egg transport
○ Yes, after each production cycle
○ No

105. Is the material/machinery used for egg collection (egg belts, belt brushes and other egg handling equipment) regularly cleaned? *(required)*

Select one option.
○ Yes, after each outgoing egg transport
○ Yes, after each production cycle
○ No
If "No" is chosen, go to question 107.

106. Is the material/machinery used for egg collection (egg belts, belt brushes and other egg handling equipment) regularly disinfected? *(required)*

Select one option.
○ Yes, after each outgoing egg transport
○ Yes, after each production cycle
○ No

107. Are the feeding systems (storage bins, augers, hoppers and chain feeders) cleaned and disinfected after each production cycle? *(required)*

Select one option.
○ Yes, cleaned and disinfected after each production cycle
○ Only cleaned after each production cycle
○ Cleaned but not after each production cycle (occasionally)
○ Never

108. Is there a feed silo/ storage and is it cleaned (and disinfected) on the inside? *(required)*

Select one option.
○ Yes, cleaned and disinfected after each production cycle
○ Only cleaned after each production cycle
○ Cleaned and disinfected but not after each production cycle (occasionally)
○ Cleaned and disinfected, but not after each production cycle. Other frequency (e.g. yearly)
○ Never

109. Is the drinking water system properly cleaned and disinfected both on the in- and outside after each production cycle? *(required)*

Select one option.
○ Always
○ Sometimes
○ Never

110. How long (in weeks) does the sanitary break after each production cycle last? *(required)*

Select one option.
○ More than 4 weeks
○ Between 1 and 4 weeks
○ Less than 1 week

L. Egg management

111. Are the eggs collected in the poultry house itself (in the same room) or are they collected in a seperated room close to the poultry house (connection with for example an automatic belt)? *(required)*

Select one option.
○ In a separated room next door
○ In the poultry house itself

112. Do you have a fully automatic egg collection system or are people manually doing the collection and/or transportation of the eggs to the egg collection room? *(required)*

Select one option.
○ There is a fully automatic system
○ Manual collection and/or transportation of the eggs by farmworkers
If "There is a fully automatic system" is chosen, go to question 114.

113. Do the farmworkers, who collect and/or transport the eggs to the sorting/packaging room, take strict preventive / hygienic measures (e.g. hand disinfection) between the egg sorting/packaging room and the poultry houses? *(required)*

Select one option.
○ Always
○ Sometimes
○ Never

114. Are the farmworkers active in the poultry houses, also working in the egg collecting room? *(required)*

Select one option.
○ Never
○ Sometimes
○ Always

115. Are dirty, cracked and/or broken eggs removed and handled separately? *(required)*

Select one option.
○ Yes
○ No

116. What type of egg trays are used to transport eggs? *(required)*

Select one option.
○ Disposable
○ Plastic
○ Cardboard (reused one/multiple times)
If "Disposable" is chosen, go to question 119.

117. Are the egg trays regularly cleaned? *(required)*

Select one option.
○ Yes, after each outgoing egg transport
○ Yes, after each production cycle
○ No
If "No" is chosen, go to question 119.

118. Are the egg trays regularly disinfected? *(required)*

Select one option.
○ Yes, after each outgoing egg transport
○ Yes, after each production cycle
○ No

119. Are hatching eggs being disinfected at the breeder farm? *(required)*

Select one option.
○ Yes
○ No
